# Colorimetric Detection of *Plasmodium vivax* in Urine Using MSP10 Oligonucleotides and Gold Nanoparticles

**DOI:** 10.1371/journal.pntd.0005029

**Published:** 2016-10-05

**Authors:** Yossef Alnasser, Cusi Ferradas, Taryn Clark, Maritza Calderon, Alejandro Gurbillon, Dionicia Gamboa, Uri S. McKakpo, Isabella A. Quakyi, Kwabena M. Bosompem, David J. Sullivan, Joseph M. Vinetz, Robert H. Gilman

**Affiliations:** 1 Johns Hopkins Bloomberg School of Public Health, Baltimore, Maryland, United States of America; 2 Universidad Peruana Cayetano Heredia, Lima, Peru; 3 King Saud University, Riyadh, Saudi Arabia; 4 Section of Emergency Medicine, Department of Medicine, Louisiana State University Health Sciences Center, New Orleans, Louisiana, United States of America; 5 School of Public Health and Noguchi Memorial Institute for Medical Research, University of Ghana, Legon, Accra, Ghana; 6 University of California San Diego, San Diego, California, United States of America; Universidad Autónoma de Yucatán, MEXICO

## Abstract

*Plasmodium vivax* is the most prevalent cause of human malaria in the world and can lead to severe disease with high potential for relapse. Its genetic and geographic diversities make it challenging to control. *P*. *vivax* is understudied and to achieve control of malaria in endemic areas, a rapid, accurate, and simple diagnostic tool is necessary. In this pilot study, we found that a colorimetric system using AuNPs and MSP10 DNA detection in urine can provide fast, easy, and inexpensive identification of *P*. *vivax*. The test exhibited promising sensitivity (84%), high specificity (97%), and only mild cross-reactivity with *P*. *falciparum* (21%). It is simple to use, with a visible color change that negates the need for a spectrometer, making it suitable for use in austere conditions. Using urine eliminates the need for finger-prick, increasing both the safety profile and patient acceptance of this model.

## Author Summary

To control malaria, there is an urgent need for applying innovative diagnostics and new technologies. Nanoparticles can augment detection of malaria at lower parasite levels while providing fast and simple methodology. Novel use of MSP10 and gold nanoparticles to identify *Plasmodium vivax*’s DNA in urine can be utilized as screening tool with global application potentials. The proposed test could impact the control of the most common species of malaria in low resource settings as it could present a simple, fast, cheap and easy to interpret test. Furthermore, utilizing urine instead of blood eliminates the need for finger-prick which would increase safety profile and likely increase participation rate in mass screening programs.

## Introduction

Malaria is the most common infectious disease in the tropics and subtropics [1]. Currently, *P*. *vivax* is endemic across Asia, the South Pacific, North Africa, Middle East, and South and Central America [2], and has recently reappeared in regions where it had previously been eradicated, including North America and Europe [3]. Currently, an estimated 2.9 billion people live at risk of *P*. *vivax* infection [4]. Research in malaria has been primarily focused on *P*. *falciparum*, the most fatal form of malaria. However, *P*. *vivax* can also cause severe illness with serious complications and costs, especially in children, in whom it has a major impact on growth [5–7].

Relying on microscopic identification of malaria species jeopardizes malaria control due to its limitations [8,9]. The WHO recognizes a need for rapid, accurate, and easy diagnostic tools in order to control malaria and need for such a test is mounting in developing countries [10]. Currently available rapid diagnostic tests (RDTs) are controversial due to their sensitivity and specificity, and differentiate poorly between plasmodium species [11–13].

With the expanding use of nanotechnology in the biomedical arena, nanoparticles can play a role in low cost, innovative diagnostics [14]. Gold nanoparticles aggregate and change their color from red to purple-blue upon exposure to single stranded DNA in aqueous solution while double stranded DNA stabilize them to preserve their red color and thus present an opportunity to develop a fast and easily interpreted diagnostic test [15–20].

Merozoite Surface Protein 10 (MSP10) is an immunogenic protein encoded by a single copy gene (in *P*. *falciparum*, GenBan Accession PF3D7 0620400; in *P*. *vivax*, PVX_114145) which is expressed in the asexual blood stages of *Plasmodium falciparum* and *P*. *vivax* [21]. One of at least 10 epidermal growth factor domain-containing proteins, the role of MSP10 in the biology of *Plasmodium* parasites has yet to be determined. *Plasmodium* MSP10 proteins have been identified as being subject to positive selection for amino acid-changing polymorphisms at the population genomic level [22,23]. Population genomics studies identify signatures of global dispersal and drug resistance in *Plasmodium vivax* [24].

Blood based tests can discourage screening both because of the pain associated with finger-prick and because of social and cultural beliefs about blood sampling. Less painful, more culturally sensitive, and safer tools for malaria diagnosis should encourage participation in mass screening programs and improve public health [25]. Urine contains circulating *P*. *vivax* DNA in detectable quantities [26–28] and can therefore serve as a less invasive and more acceptable sample for malaria screening and diagnosis. Also, urine contains less interfering proteins and inhibitors than blood which allows easier DNA extraction [29]. Furthermore, urine provides lower risks to healthcare personnel, with reliable amounts of malaria DNA found in urine despite being substantially lower than blood samples [30]. Additionally, urine color is not expected to obscure color change of gold nanoparticles.

In this pilot study, we tested the hypothesis that a colorimetric system using gold nanoparticles and MSP10 DNA detection in urine would be useful as a safe diagnostic and surveillance tool for *P*. *vivax*. Such a tool is needed for improving malaria control in the endemic setting.

## Materials and Methods

### Chemicals and Reagents

Citrate reduced gold 15nm nanoparticles, and KCl were purchased from Sigma Aldrich (St. Louis, MO, United States). PBS was obtained from Invitrogen (Grand Island, NY, United States). NaCl and NaOH were acquired from Merck Millipore (Kenilworth, NJ, United States).

### MSP10 Oligonucleotides

The two MSP10 oligonucleotides utilized in this study were a generous gift from Professor Mirko Zimic (Universidad Peruana Cayetano Heredia, Lima, Peru) and designed by Dr. Joseph Vinetz (University of California San Diego, United States). Crafted to represent the C-Terminal segment of MSP10, the first oligonucleotide has a sequence of 5´CACCATGGAACAGTTTATCCTGAAGAC3'. The other oligonucleotide was used as a representative of the N-terminal segment of MSP10. It has a sequence of 5´AGCCATGGAACGTGCTAAGTGCAACA3’.

### Urine Samples

Archived urine samples positive for *P*. *vivax* and *P*. *falciparum* were collected from Iquitos in Peru and Ghana, respectively. Negative control urine samples were collected from volunteers who were blood smear negative in Iquitos, Peru and in Ghana, as well as in Lima, Peru, which is a non-endemic site. All urine samples were collected by clean catch procedures. Ghana urine samples were pelleted in the field and shipped on dry ice, pH was adjusted and samples were refrozen at -80°C as described earlier [31]. Peru urine samples were stored initially at -20°C prior to freezing at -80°C (Table 1). Peru’s urine samples were stored for 8 months while all Ghana samples were stored for more than one year.

**Table 1:** Urine samples were collected from three different sites representing Africa and South America.

Urine Sample	Origin	Quantity
Positive for *P*. *vivax*	Iquitos, Peru	31
Positive *for P*. *falciparum*	Ghana	14
South American Controls	Iquitos, Peru	8
South American Controls	Lima, Peru	28
African Controls	Ghana	9

### Blood Smears

During the epidemiological surveys on the communities, the field microscopist reports whether a slide is positive or negative, and identifies the species, *P*. *vivax* and *P*. *falciparum*. They read 300 microscopy fields before the slide is reported as negative.

In the laboratory, a second reader (an experienced microscopist working for research projects for more than 15 years) read the slide to report species and parasite density assuming a white blood cell count of 6,000/μl. The research microscopist read until 500 microscopy fields, before the slide is reported as negative.

For quality control, 10% randomly selected slides (positive and negative) were reexamined by two blinded, expert microscopists at a reference laboratory in Loreto from Peru’s Ministry of Health. From the quality control examinations, the level of concordance varies between 98–100% for species and parasite density.

### Colorimetric System

Urine was thawed at 25°C. Once urine was at room temperature, dipsticks were carried out to determine urine pH and the presence of protein. Each urine sample was centrifuged at 15,000 rpm for 5 minutes to remove sediments and then filtered using a 0.2 mm membrane (Minisart, Bohemia, NY, United States) to remove possible confounding particulates. The urine samples were diluted 1:16 with PBS. Diluted samples’ pH was adjusted to reach ≈ 6.4 using pH meter, and HCl and NaOH solutions. 50 uL of each diluted urine sample was heated at 95°C for 30 seconds using a thermocycler. Samples were cooled at room temperature for 10 minutes and 10 uL of either C-Terminal or N-Terminal MSP10 oligonucleotides and 20 uL of 0.25 M NaCl were added. The sample was heated at 59°C for two minutes and allowed to cool to room temperature for ten minutes. Finally, 50 uL of citrate reduced AuNPs were added. Two minutes later, the system was read visually and by spectrophotometer.

### Ethics Statement

Urine and blood were collected for previous studies that were approved by institutional review boards of Universidad Peruana Caytano Heredia and University of Ghana, respectively. Written informed consents were obtained prior to storing samples as anonymous and unidentified.

## Results

### Color Change and Detection of *P*. *vivax*

**Fig 1:**
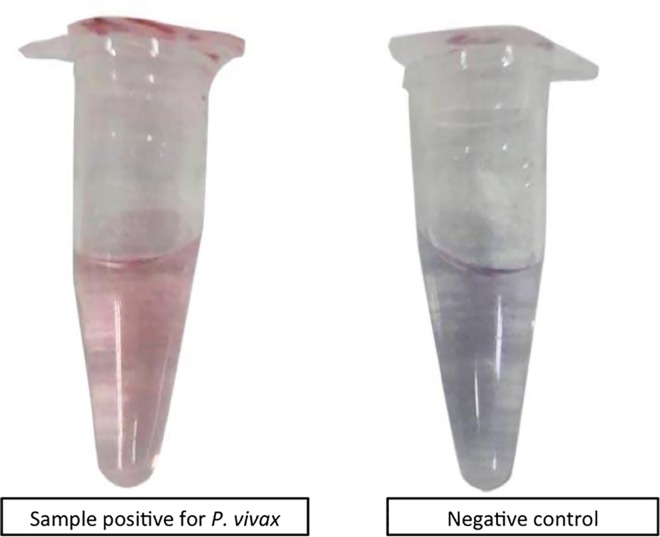
Urine from *P*. *vivax* negative volunteers turned AuNPs blue due to lack of targeted MSP10 DNA while positive *P*. *vivax* urine was able to form DS DNA and stabilized AuNPs to stay red in color.

**Fig 2:**
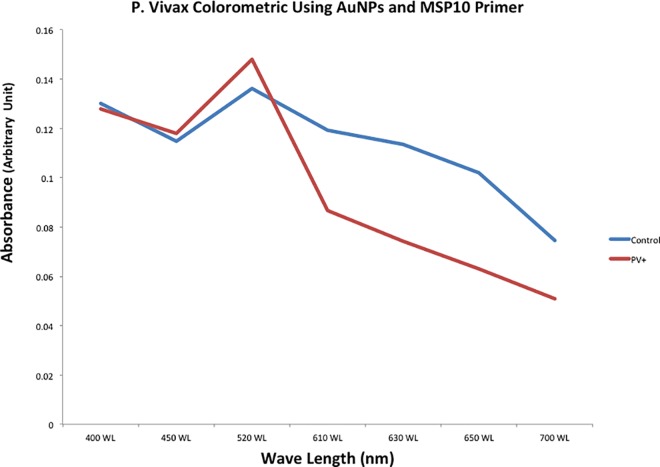
The newly formed MSP10 double stranded DNA in urine was able to stabilize AuNPs to stay red in color. *P*. *vivax* negative urine turned AuNPs’ color to blue which was detected by Spectrophotometer as color switches from red at 520 wavelength *(P*. *vivax* positive) into purple-blue at 610 wavelength (*P*. *Vivax* negative).

Up to 84% of *P*. *vivax* positive samples stabilized the gold nanoparticles and maintained a red color while 97% of negative controls induced aggregation and allowed color change to purple-blue. This color change was distinctly distinguished by naked eye (Fig 1). Additionally, the color difference was well defined by spectrophotometer, with positive samples at wavelengths of 520 and negative samples exhibiting wavelengths of 610–630 (Fig 2).

### Sensitivity

The colorimetric system was able to detect *P*. *vivax* with variable sensitivity. The sensitivity was dependent on which segment of MSP10 was used. The N-terminal segment distinguished 26 of 31 positive samples (84%) while the C-terminal segment distinguished only 20 of 31 samples (65%).

### Specificity

**Fig 3:**
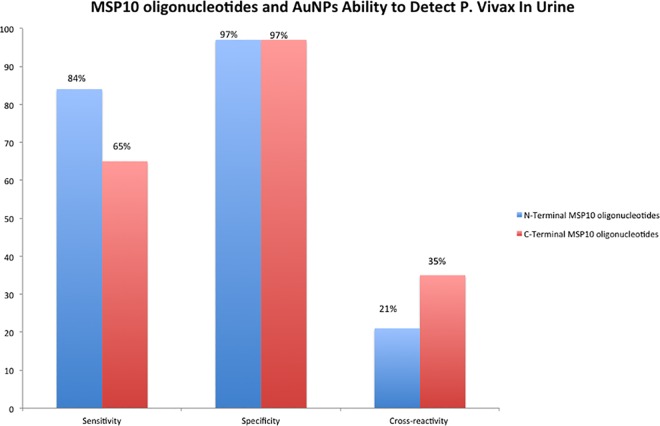
N-Terminal MSP10 oligonucleotide has higher sensitivity, similar specificity and lower cross-reactivity in comparison to C-Terminal segment of MSP10 DNA in detecting *P*. *vivax* in urine using AuNPs.

Both the N-terminal and C-terminal segments of MSP10 had an overall specificity of 97% in urine, with only one false positive out of 45 control samples. The false positive was from a laboratory control in Lima (Fig 3).

### Parasitemia Level

Parasitemia level was determined by blood smears collected at the same time as the urine samples. Data were shared after running the colorimetric system on urine to minimize investigator bias. There was no correlation between parasitemia level in blood and false negativity. The lowest parasitemia level observed in blood smear that also had a positive colorimetric test was 12 parasite/uL. However, there were two false negative samples with average parasitemia level in blood of 2510 parasite/uL (Table 2).

**Table 2:** Blood parasitemia levels showed poor correlation with both oligonucleotides in regard to their ability to detect *P*. *vivax* in urine using AuNPs.

*P*. *vivax* Urine Sample	Age of study participant (in years)	N-Terminal Oligoneclutide	C-Terminal Oligoneclutide	Parasite Count by Microscope—Parasitos Asexuales (par/ul)
1	56	red	Red	840
2	22	red	Red	24
3	24	red	purple-blue	36
4	37	red	Red	5310
5	51	red	Red	328
6	50	red	Red	360
7	40	red	purple-blue	36
8	31	red	Red	12
9	48	red	Red	720
10	18	red	purple-blue	1166
11	21	red	purple-blue	1080
12	68	purple-blue	purple-blue	420
13	24	red	Red	7620
14	42	red	Red	1680
15	55	red	Red	24
16	18	red	purple-blue	24
17	13	red	purple-blue	24
18	35	purple-blue	purple-blue	2350
19	40	purple-blue	purple-blue	2670
20	30	red	red	360
21	57	red	red	1098
22	54	red	purple-blue	600
23	26	purple-blue	purple-blue	24
24	68	red	Red	60
25	22	red	Red	108
26	28	red	red	12
28	50	red	red	2610
29	17	purple-blue	red	360
30	32	red	Red	5520
31	44	red	red	4470

### Cross-Reactivity with *P*. *falciparum*

N-Terminal MSP10 oligonucleotide had a lower cross-reactivity (21%). C-Terminal MSP10 oligonucleotides showed high cross-reactivity with *P*. *falciparum* in urine utilizing colorimetric system (36%) (Table 3).

**Table 3:** MSP10 N-Terminal oligonucleotide and AuNPs were able to detect *P*. *vivax* in urine with higher sensitivity, specificity and lower cross-reactivity than C-Terminal oligonucleotide of MSP10.

	Origin	MSP10 N-Terminal oligonucleotide Detection	MSP10 C-Terminal oligonucleotideDetection
Urine Samples Positive for *P*. *vivax*	Iquitos, Peru	26	20
		(26/31: 84%)	(20/31: 65%)
Urine Samples Positive for *P*. *falciparum*	Ghana	3	5
		(3/14: 21%)	(5/14: 36%)
South American Negative Urine Samples	Peru	1	1
		(1/36: 3%)	(1:36: 3%)
African Negative Urine Samples	Ghana	0	0
		(0/9: 0%)	(0/9: 0%)

### Duration and Cost

It took an average of 45 minutes from collecting urine to reading the test. Cost of the raw materials for each test is $0.20.

## Discussion

Currently available RDTs suffer from limited sensitivity, specificity, genetic instability, the inability to differentiate *Plasmodium* species, expense, and limitation in sample types (blood only) [11–13]. Blood tests also cannot be performed in many field settings since expertise, temperature control, storage, and laboratory equipment are unavailable, [11]. Urine has been reported as an accessible and reliable source of malaria DNA with lower detection threshold than blood; however, it has lower levels of detectable antigen and current RDTs have a minimum detection level of 100 parasites per microliter [26,32]. However, urine contains smaller double stranded DNA of 150-to 250-nucleotide size that can interact with nanoparticles and still be utilized as a valid source of microorganisms’ DNA [28,33].

*P*. *vivax* presents unique diagnostic challenges because of its genetic and geographic diversities [34,35]. The emergence of sequencing technologies and malaria sequences is providing us with a greater understanding of conserved regions, which must be targeted for broad-applicability of diagnostic tests [36]. Merozoite Surface Protein 10 (MSP10) is one of the asexual stage proteins of *P*. *vivax* linked to erythrocyte invasion [37]. It has two prominent EGF-like domains at the C-terminus, which are highly conserved and carry close homogeneity among all *Plasmodium* species [38,39].

We used two segments in our study. The N terminal segment showed superior sensitivity and equal specificity when compared to the C terminal segment. It could be due to its higher adenine and guanine contents as they both have higher adsorption rate to AuNPs’ surface [40,41].

The sensitivity of MSP10 oligonucleotides in detecting DNA in urine samples is dependent on quality of urine. Stored urine is known to have less DNA than fresh urine and age could play an important role in determining sensitivity of MSP10 oligonucleotides [28].

MSP10 has little similarity among *Plasmodium* species apart from the two EGF-like domains [21], which could explain lower cross-reactivity of N-terminal segment with *P*. *falciparum*. To be able to lower cross-reactivity, optimization of MSP10 oligonucleotides is required in future work.

Currently, most commercially and widely used malaria RDTs employ monoclonal antibodies to identify histidine-rich protein two (HRPII) [32]. Although HRPII based RDTs reported variable sensitivity and specificity, HRPII was subject to genetic and geographical diversities along with gene polymorphism [42]. Additionally, monoclonal antibodies against HRPII might cross react with other proteins [42]. In Peru, approximately 30% of *P*. *Falciparum* had HRPII gene deletions, which might lead to false negative RDTs results [43]. Additionally, other RDTs employed malaria markers that have been subject to controversy. Aldolase, an isoenzyme widely used to diagnose *P*. *falciparum* and *P*. *vivax*, was criticized for its low sensitivity and genetic variability in diagnosing *P*. *vivax* [44]. *P*.*vivax’s* lactate dehydrogenase (pLDH), another common antigen used in malaria RDTs, was found to be affected by its gene polymorphism [45]. Furthermore, pLDH requires whole blood sampling and declines very fast, with clearance of asexual parasitemia [45]. This study provides evidence that MSP10 DNA can serve as a marker for malaria at a global scale, and may deliver innovative tools to aid in malaria control.

In developing other diagnostic tests, nanoparticles have been utilized both for concentrating and for detecting samples with low concentration of target molecules in austere settings [46–48]. Jeon et al in 2013 found that AuNPs could be used to recognize *P*. *vivax* DNA in diluted blood samples using pLDH with detection levels as low as 74 parasites/uL [46]. We were unable to determine our detection level due to lack of correlation between *Plasmodium* species’ blood and urine DNA levels, which has been previously reported [30]. Despite our ability to detect a positive urine sample in a patient with low blood parasitemia, we had false negative samples with very high blood parasitemia levels indicating poor correlation between our test and parasitemia levels in blood.

In spite of lack of correlation between blood and urine DNA, this test has many other advantages. With further simplification of the process, it has potential to represent a simple, rapid test that does not require extensive lab skills, which will make it suitable as point of care test for low resource settings [49].

### Conclusion

To our knowledge, this test is the first RDT utilizing urine samples rather than blood and employing nanoparticles. The colorimetric assay using AuNPs and MSP10 oligonucleotides to detect *P*. *vivax* in urine holds potentials to provide a safe, simple, rapid, and cheap tool to diagnose one of the most common form of malaria. Innovative use of MSP10 as a marker for *P*. *vivax* has potential for global application in mass screening programs.

## Supporting Information

S1 File STARD Flow Diagram. (DOCX)

S2 File STARD Checklist. (DOCX)
