# Correction: Colorimetric Detection of *Plasmodium vivax* in Urine Using MSP10 Oligonucleotides and Gold Nanoparticles

**DOI:** 10.1371/journal.pntd.0005143

**Published:** 2016-11-09

**Authors:** Yossef Alnasser, Cusi Ferradas, Taryn Clark, Maritza Calderon, Alejandro Gurbillon, Dionicia Gamboa, Uri S. McKakpo, Isabella A. Quakyi, Kwabena M. Bosompem, David J. Sullivan, Joseph M. Vinetz, Robert H. Gilman

In the Funding section, the grant number from the funder NIAID/ICEMR Amazonia is listed incorrectly. The correct grant number is: U19AI089681.
