# Supplementary material for: Colorimetric Detection of Plasmodium vivax in Urine Using MSP10 Oligonucleotides and Gold Nanoparticles
Source: PLoS Negl Trop Dis. 2016 Oct 5;10(10):e0005029. doi: 10.1371/journal.pntd.0005029 (PMC5051960; doi:10.1371/journal.pntd.0005029)
Supplement: S1 File — (DOCX) [file pntd.0005029.s001.docx]

STARD diagram to report flow of participants through the study
